# Depletion of peroxiredoxin II promotes keratinocyte apoptosis and alleviates psoriatic skin lesions via the PI3K/AKT/GSK3β signaling axis

**DOI:** 10.1038/s41420-023-01566-z

**Published:** 2023-07-27

**Authors:** Ying-Hao Han, Lin Feng, Seung-Jae Lee, Yong-Qing Zhang, Ai-Guo Wang, Mei-Hua Jin, Hu-Nan Sun, Taeho Kwon

**Affiliations:** 1 College of Life Science and Technology, Heilongjiang Bayi Agricultural University, 163319 Daqing, Heilongjiang P.R. China; 2 Functional Biomaterial Research Center, Korea Research Institute of Bioscience and Biotechnology, Jeongeup-si, Jeonbuk 56212 Republic of Korea; 3 Department of Applied Biological Engineering, KRIBB School of Biotechnology, University of Science and Technology, Daejeon, 34113 Republic of Korea; 4 Laboratory Animal Center, Dalian Medical University, 116041 Dalian, P.R. China; 5 Primate Resources Center, Korea Research Institute of Bioscience and Biotechnology (KRIBB), Jeongeup-si, Jeonbuk 56216 Republic of Korea; 6 Department of Functional Genomics, KRIBB School of Bioscience, University of Science and Technology, Daejeon, 34113 Republic of Korea

**Keywords:** Stress signalling, Skin diseases

## Abstract

Psoriasis is a chronic, systemic immune-mediated disease caused by abnormal proliferation, decreased apoptosis, and over-differentiation of keratinocytes. The psoriatic skin lesions due to abnormal keratinocytes are closely associated with ROS produced by inflammatory cells. Peroxiredoxin II (Prx II) is an efficient antioxidant enzyme, which were highly expressed in skin tissues of psoriasis patient. However, the detailed mechanical functions of Prx II on psoriatic skin remain to be elucidated. Present study showed that depletion of Prx II results in alleviation of symptoms of IMQ-induced psoriasis in mice, but no significant differences in the amounts of serum inflammatory factors. Prx II-knockdown HaCaT cells were susceptible to H_2_O_2_-induced apoptosis mediated by Ca^2+^ release from the endoplasmic reticulum through 1,4,5-triphosphate receptors (IP3Rs), the PI3K/AKT pathway and phosphorylated GSK3β (Ser9) were significant downregulated. Additionally, significantly reduced sensitivity of Prx II-knockdown HaCaT cells to apoptosis was evident post NAC, 2-APB, BAPTA-AM, SC79 and LiCl treated. These results suggest that Prx II regulated apoptosis of keratinocytes via the PI3K/AKT/GSK3β signaling axis. Furthermore, treatment with the Prx II inhibitor Conoidin A significantly alleviated psoriatic symptoms in IMQ model mice. These findings have important implications for developing therapeutic strategies through regulate apoptosis of keratinocytes in psoriasis, and Prx II inhibitors may be exploited as a therapeutic drug to alleviate psoriatic symptoms.

## Introduction

The skin disease psoriasis may occur as a consequence of several factors including infectious microorganisms, and environmental, immunological, and genetic factors [1]. The appearance of psoriatic skin lesions characterized by erythema as well as painful and itchy hardened scaly skin is the most common presentation [2–4]. The appearance of psoriatic lesions on normal skin drastically modifies visual appearance, and thereby results in deterioration of a patient’s quality of life. In recent years, psoriasis has witnessed the emergence of improved treatment strategies. Despite these advances, however, it remains a manageable but incurable disease. A better comprehension of the pathogenic mechanisms responsible for disease onset and progression, will therefore, help in developing effective therapeutic options.

Abnormal proliferation, over differentiation, and decreased apoptosis of keratinocytes are the main cellular events that lead to the occurrence and development of psoriasis [5–7]. Neutrophils at lesions sites have strong antibacterial action, including degranulation and production of reactive oxygen species (ROS) [8]. Excess production of ROS induces oxidative modification of macromolecules in epidermal keratinocytes, inhibits various protein functions, and ultimately affects cell viability [9]. However, the ubiquitous presence of antioxidant enzymes that reduce or counteract cellular peroxidation induced by ROS, including glutathione (GSH), superoxide dismutase (SOD), catalase (CAT) (337), glutathione peroxidase (GPX), and peroxiredoxin (Prxs) have been reported in a variety of cells [10–12]. The effects exerted by these enzymes reduce ROS-mediated cellular damage in epidermal keratinocytes, which is closely associated with abnormal proliferation and decreased apoptosis. Consequently, Prxs may play an important regulatory role in the development and severity of psoriatic skin lesions.

Comparative studies of the proteome of normal and psoriatic skin have revealed differences in proteins that maintain the redox balance between the two. The expression of Peroxiredoxin 2 (Prx II), a member of the Peroxiredoxin (Prxs) family, was upregulated in psoriatic skin tissue [13]. Prx II function as peroxide reductase, and is widely distributed among various cell types. This protein is responsible for the removal of low levels of ROS, and consequently play an important role in cellular proliferation and apoptosis [14–16]. We therefore speculated on the possible regulatory role of Prx II in psoriatic skin lesions. The present study examined this hypothesis by analyzing the effect of imiquimod (IMQ) that induces psoriatic skin lesions in Prx II-depleted skin tissue. The study also explored in vitro the regulatory mechanisms involving Prx II in these lesions.

## Results

### Amelioration of symptoms and reduction of epidermal thickness of IMQ induced psoriatic skin lesions in Prx II-knockout mice

**Fig. 1:**
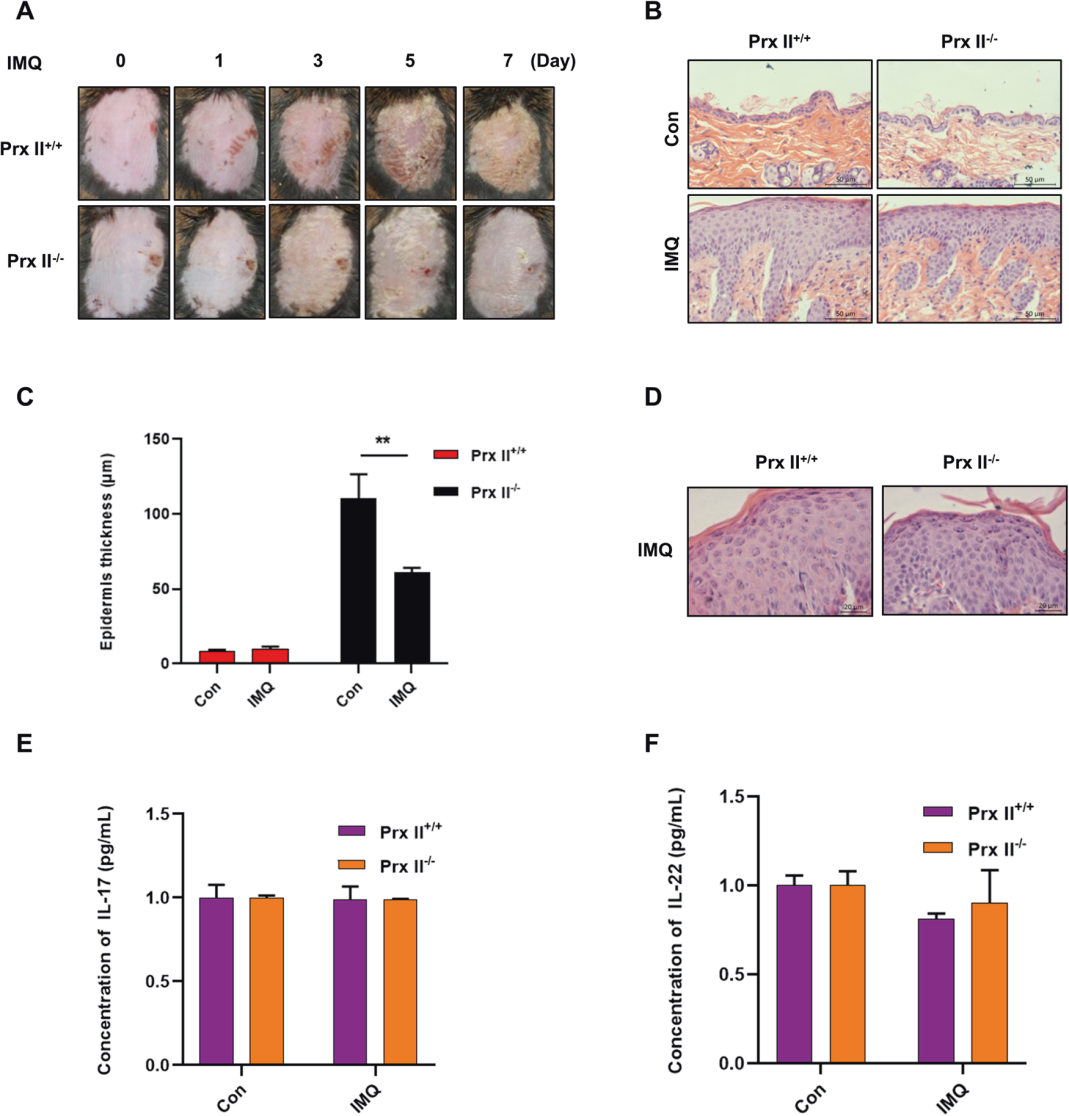
Effect of Prx II on psoriasis in mice. **A** Damage to skin on the back of mice post IMQ treatment in Prx II^+/+^ mice and Prx II^−/−^ mice (*n* = 6 in each group). **B** Histological changes in mouse skin as seen by H&E staining. **C** Skin thickness is reduced in Prx II^−/−^ mice compared to Prx II^+/+^ mice. The data are represented by the mean ± SD (*n* = 6). **D** Histological evaluation reveals the presence of microabscesses. **E**, **F** Levels of serum L-17 and IL-22 as measured by enzyme-linked immunosorbent assay (ELISA). The data are represented by the mean ± SEM of three separate samples. (**p < 0.01).

The regulatory role of Prx II was studied in psoriasis models that were generated in Prx II^+/+^ and Prx II^−/−^ mice by the application of IMQ on their backs. The degree of skin damage on the backs of Prx II^−/−^ mice was found to be significantly lesser (Fig. 1A), with significantly thinner skin (Fig. 1B, C), than that on the backs of Prx II^+/+^ mice on the seventh day. Although microabscesses were observed, and neutrophils were seen to gather in the upper part of the spinous and granular layers, no significant differences were observed between the epidermis of the two mice (Fig. 1D). Further, no significant differences in the amounts of serum inflammatory factors, including IL-17 and IL-22, were observed between the two mice post application of IMQ (Fig. 1E, F). These results suggest that depletion of Prx II results in alleviation of skin lesions in IMQ-induced psoriasis in mice.

### Sensitivity of Prx II-knockdown HaCaT cells to H_2_O_2_ cytotoxicity

**Fig. 2:**
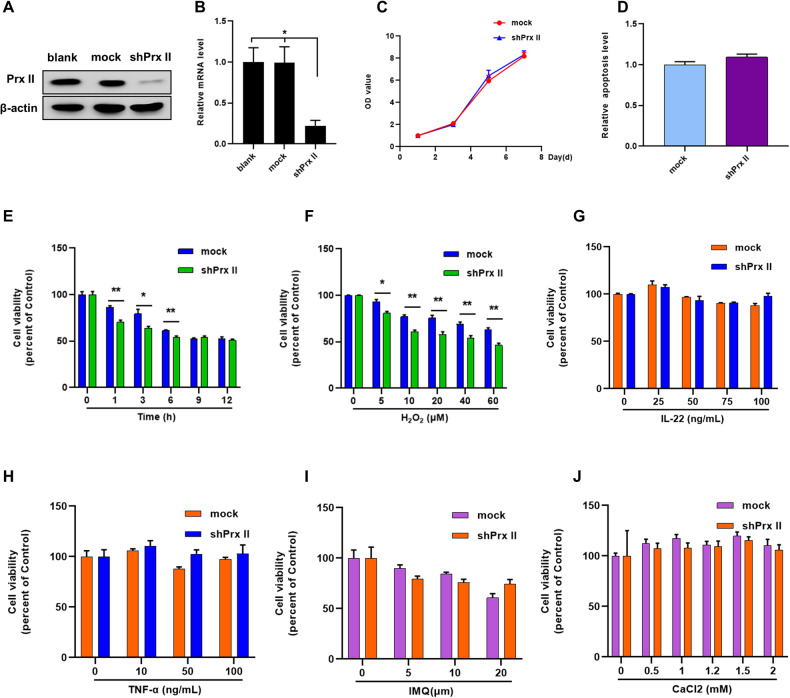
Effect of Prx II on HaCaT cells viability post H_2_O_2_ stimulation. **A**, **B** Expression levels of Prx II (Protein and mRNA) in mock and shprx II HaCaT cells as assessed by western blotting and real time PCR. **C** Measurement of mock and shprx II HaCaT cells viability by MTT. **D** Detection of apoptosis by assessing fluorescent intensity of Annexin V-PE by flow cytometry. **E**, **F** Time and H_2_O_2_ concentration-dependent cell viability of mock and shprx II HaCaT cells. **G**–**J** Cell viability of mock and shprx II HaCaT cells post IMQ, CaCl_2,_ IL-22, and TNF-α treatment. The data are represented by the mean ± SEM of three separate samples. (*p < 0.05, **p < 0.01).

Efficacious knockdown of the Prx II gene in HaCaT cells required successful intracellular vector transfer. The possible interference of the vector with Prx II gene expression was analyzed by comparing Prx II protein and mRNA levels by western blotting and real time PCR in both control (mock) and Prx II-knockdown (sh Prx II) cells (Fig. 2A, B), which revealed significantly lower levels of Prx II in the latter. A concomitant significant effect on cell growth and activity was not evident (Fig. 2C, D). Previous studies have demonstrated the importance of apoptosis in the treatment of psoriasis in HaCaT cells [6]. This was assessed by treating HaCaT cells with H_2_O_2_, IMQ, CaCl_2_, and inflammatory factors such as IL-22 and TNF-α, followed by the detection of apoptosis by the MTT assay. The results revealed that H_2_O_2_ significantly induced apoptosis in Prx II-knockdown HaCaT cells in a time- and concentration-dependent manner (Fig. 2E, F); however, no significant effect was evident in the other treatment groups (Fig. 2G–J).

### Induction of mitochondrial dependent apoptosis by H_2_O_2_ in Prx II-knockdown HaCaT cells

**Fig. 3:**
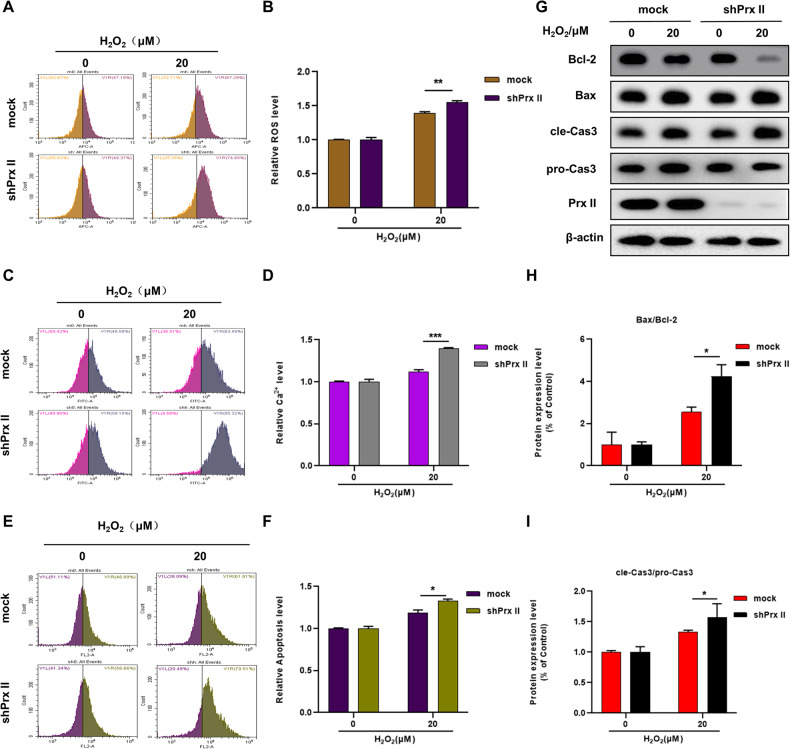
Effect of Prx II on H_2_O_2_ induced ROS and Ca^2+^ accumulation and mitochondria dependent apoptosis in HaCaT cells. **A**, **B** Detection and quantitation of intracellular ROS by flow cytometry. **C**, **D** Detection and quantitation of intracellular Ca^2+^ by flow cytometry. **E**, **F** Detection and quantification of apoptosis in mock and shPrx II HaCaT cells post H_2_O_2_ treatment by flow cytometry. **G**–**I** Analysis and quantification of expression levels of Prx II and apoptosis-related proteins including Bax/Bcl-2 and cle-Cas3/Pro-Cas3 by western blotting post 6 h treatment with H_2_O_2_ in HaCaT cells. The data are represented by the mean ± SEM of three separate samples. (*p < 0.05, **p < 0.01, ***p < 0.001).

Intracellular ROS levels in HaCaT cells were estimated post treatment with H_2_O_2_ to shed light on underlying mechanisms involved in the sensitivity of Prx II-knockdown HaCaT cells to H_2_O_2_ cytotoxicity. The results revealed significant elevation in the accumulation of ROS in Prx II knockdown cells as compared to that in control cells after H_2_O_2_ treatment (Fig. 3A, B). Moreover, the accumulation of cytoplasmic Ca^2+^ significantly elevated in Prx II knockdown HaCaT cells after H_2_O_2_ treatment (Fig. 3C, D). As expected, the level of apoptosis was significantly higher in shPrx II HaCaT cells than in mock cells (Fig. 3E, F). Further, western blot analysis was used to detect the expression levels of apoptosis related proteins, which revealed a significant downregulation of Bcl-2, and upregulation of Bax and cleaved Caspase-3 in shPrx II HaCaT cells (Fig. 3G). The Bax/Bcl-2 and cle-Cas3/pro-Cas3 ratio, which were closely associated with accumulated cytoplasmic ROS and Ca^2+^ induced apoptosis were found to be significantly increased (Fig. 3H, I). These findings demonstrate H_2_O_2_ induced mitochondrial-dependent apoptosis in Prx II-knockdown HaCaT cells.

### Reversal of H_2_O_2_ induced upregulation of intracellular ROS and Ca^2+^ levels and apoptosis in Prx II-knockdown HaCaT cells by NAC treatment

**Fig. 4:**
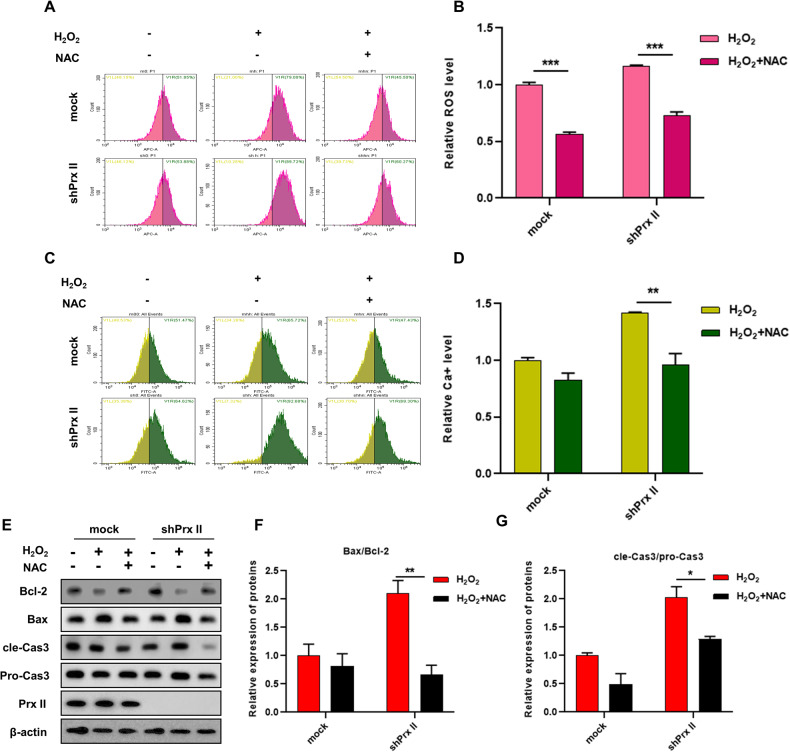
Effect of NAC treatment on ROS, Ca^2+^ accumulation and apoptosis in Prx II knockdown HaCaT cells. **A**, **B** Detection and quantitation of intracellular ROS by flow cytometry. **C**, **D** Detection and quantitation of intracellular Ca^2+^ by flow cytometry. **E**–**G** Analysis and quantification of expression levels of Bax/Bcl-2 and cle-Cas3/pro-Cas3 by western blotting post pre-treated with NAC for 30 min, followed by H_2_O_2_ treatment for 1 h. in HaCaT cells. The data are represented by the mean ± SEM of three separate samples. (*p < 0.05, **p < 0.01, ***p < 0.001).

Treatment of NAC significantly reversed the increase in ROS and Ca^2+^ levels induced by H_2_O_2_ in HaCaT cells (Fig. 4A–D). Treatment with NAC also significantly inhibited the upregulation of Bax and cle-Caspase3, while concomitantly inducing the upregulation of Bcl-2 (Fig. 4E). The Bax/Bcl-2 and cle-Cas3/pro-Cas3 ratio were found to be significantly decreased (Fig. 4F, G). These findings suggest that the reduction of intracellular ROS attenuates apoptosis induced by H_2_O_2_ in Prx II-knockdown HaCaT cells by NAC treatment.

### Reversal of apoptosis induced by H_2_O_2_ in Prx II-knockdown HaCaT cells by BAPTA-AM and 2-APB treatment

**Fig. 5:**
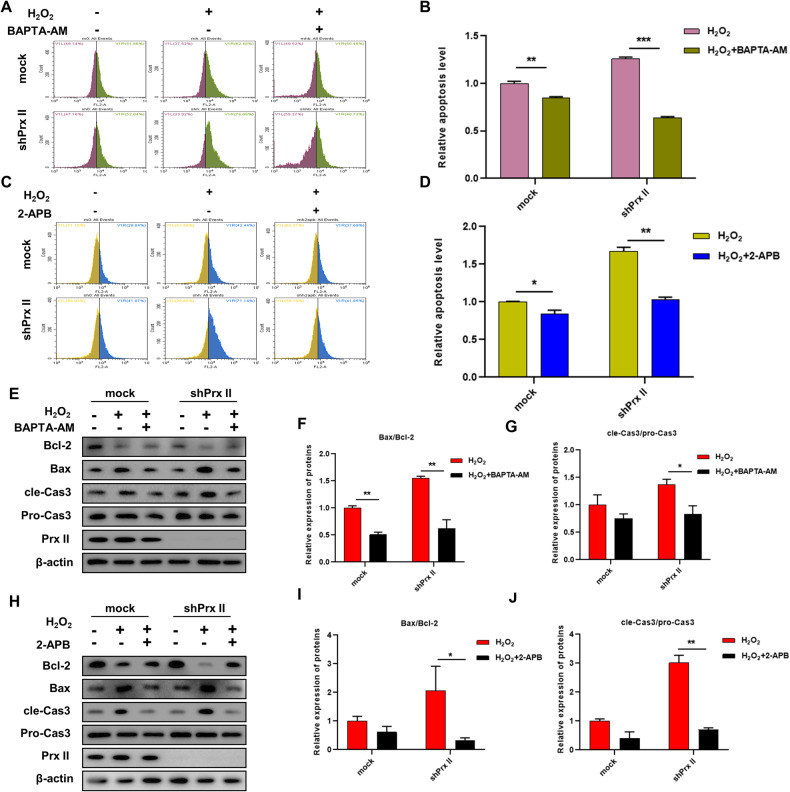
Effect of BAPTA-AM and 2-APB treatment on apoptosis in Prx II knockdown HaCaT cells. **A**, **B** Detection of apoptosis in Prx II-knockdown HaCaT cells by flow cytometry. **C**, **D** Detection of apoptosis in Prx II-knockdown HaCaT cells by flow cytometry. **E**–**G** Analysis of expression levels of Bax/Bcl-2 and cle-Cas3/pro-Cas3 by western blotting pre-treated with BAPTA-AM for 30 min, followed by H_2_O_2_ treatment for 1 h. **H**–**J** Analysis of expression levels of Bax/Bcl-2 and cle-Cas3/pro-Cas3 by western blotting pre-treated with 2-APB for 30 min, followed by H_2_O_2_ treatment for 1 h. The data are represented by the mean ± SEM of three separate samples. (*p < 0.05, **p < 0.01, ***p < 0.001).

Treatment with BAPTA-AM, a selective Ca^2+^ chelating agent significantly reversed apoptosis in Prx II-knockdown HaCaT cells induced by H_2_O_2_ treatment (Fig. 5A, B). The underlying mechanism responsible for the reversal of mitochondria-dependent apoptosis induced by H_2_O_2_, consequent to elimination of intracellular Ca^2+^ was explored by employing 2-aminoethyl diphenylborinate (2-APB), a commonly used inositol 1,4,5-trisphosphate receptor(IP3R) inhibitor [17]. Treatment with 2-APB significantly reversed apoptosis in Prx II-knockdown HaCaT cells induced by H_2_O_2_ treatment (Fig. 5C, D). Simultaneously, both BAPTA-AM and 2-APB treatment also significantly inhibited the upregulation of Bax and cle-Caspase3 induced by H_2_O_2_, and induced the upregulation of Bcl-2 in Prx II-knockdown HaCaT cells, the Bax/Bcl-2 and cle-Cas3/pro-Cas3 ratio were found to be significantly decreased (Fig. 5E–J). These results provide further evidence that the elimination of intracellular Ca^2+^ prevents apoptosis induced by H_2_O_2_ in Prx II-knockdown HaCaT cells.

### Prx II mediated H_2_O_2_-induced apoptosis in HaCaT cells via PI3K/AKT/GSK3β pathway

**Fig. 6:**
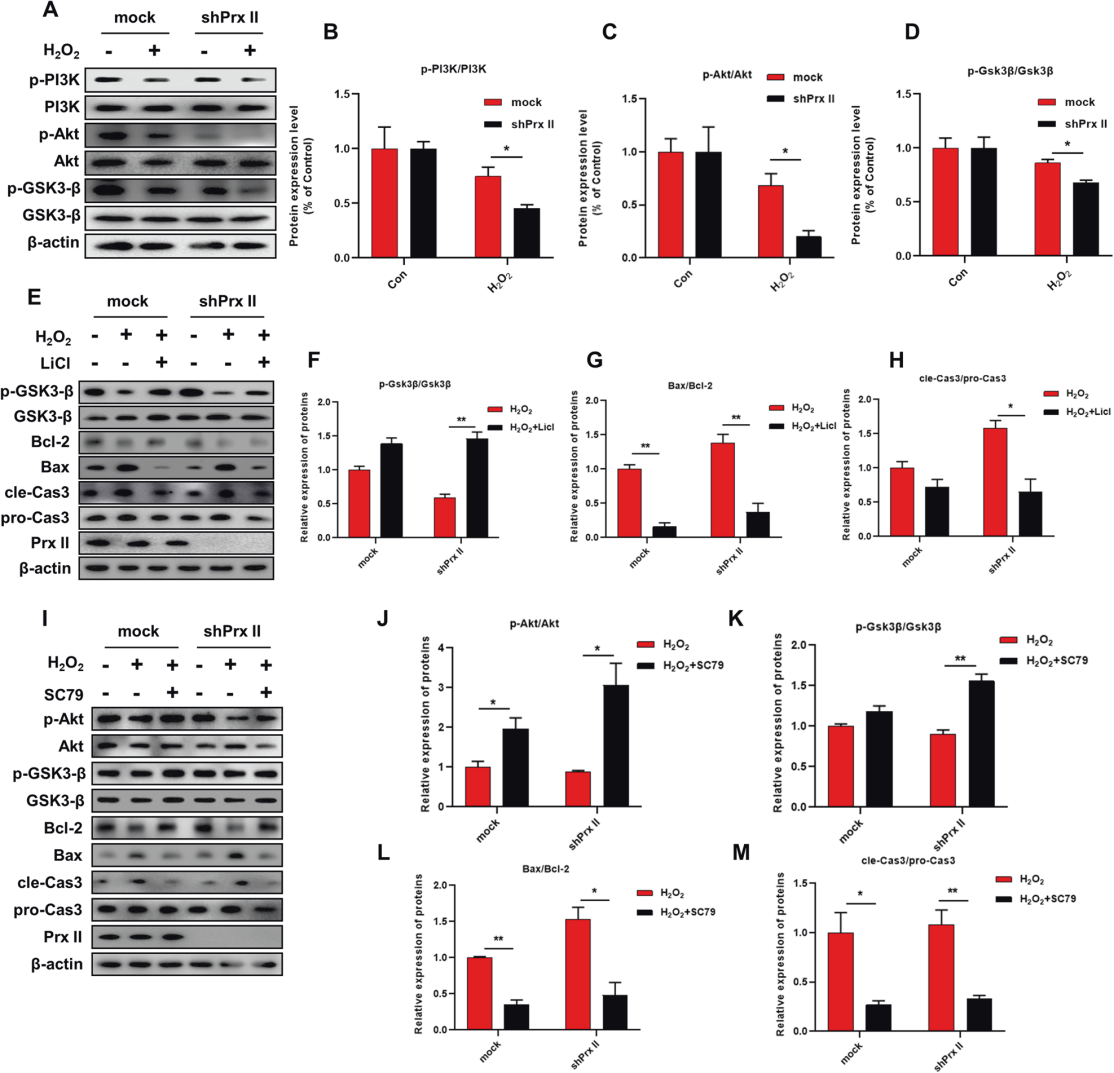
Effect of LiCl and SC79 pretreatment on PI3K/Akt/Gsk3β signaling pathway and apoptosis related protein expression in Prx II knockdown HaCaT cells. **A**–**D** Analysis of expression levels of p-PI3K/PI3K, p-Akt/Akt and p-Gsk3β/Gsk3β by western blotting by H_2_O_2_ treatment for 1 h. **E**–**H** Analysis of expression levels of p-Gsk3β/Gsk3β, Bax/Bcl-2 and cle-Cas3/pro-Cas3 by western blotting pre-treated with Licl for 1 h, followed by H_2_O_2_ treatment for 1 h. **I**–**M** Analysis of expression levels of p-Akt/Akt, p-Gsk3β/Gsk3β, Bax/Bcl-2, and cle-Cas3/pro-Cas3 by western blotting pre-treated with SC79 for 30 min, followed by H_2_O_2_ treatment for 1 h. The data are represented by the mean ± SEM of three separate samples. (*p < 0.05, **p < 0.01).

GSK3β plays a key role in regulating the activity of IP3R. phosphorylated GSK3β (Ser9) level was significantly downregulated in Prx II-knockdown HaCaT cells after H_2_O_2_ treatment, accompanied by a marked decrease in the phosphorylation levels of its upstream kinase PI3K and AKT (Fig. 6A–D). Treated with GSK3β inhibitor LiCl, the expression levels of the phosphorylated GSK3β (Ser9) was significantly upregulated, Bax and cle-Caspase3 protein were found to be significantly downregulated, and that of Bcl-2 was significantly upregulated in Prx II-knockout HaCaT cells after H_2_O_2_ treatment(Fig. 6E–H). Then treated with AKT activator SC79, the expression levels of the phosphorylated AKT, phosphorylated GSK3β (Ser9) were significantly upregulated, Bax and cle-Caspase3 protein were found to be significantly downregulated, and that of Bcl-2 was significantly upregulated in cells treated with SC79 as compared to that in control cells (Fig. 6I–M). Its shows that treatment with Licl and Sc79 can significantly reversed apoptosis signaling induced by H_2_O_2_ in Prx II-knockdown HaCaT cells. These results indicate that the Prx II mediated H_2_O_2_-induced apoptosis in HaCaT cells by regulating PI3K/AKT/GSK3β pathway.

### Improvement of symptoms of psoriasis in mice by Conoidin A treatment

**Fig. 7:**
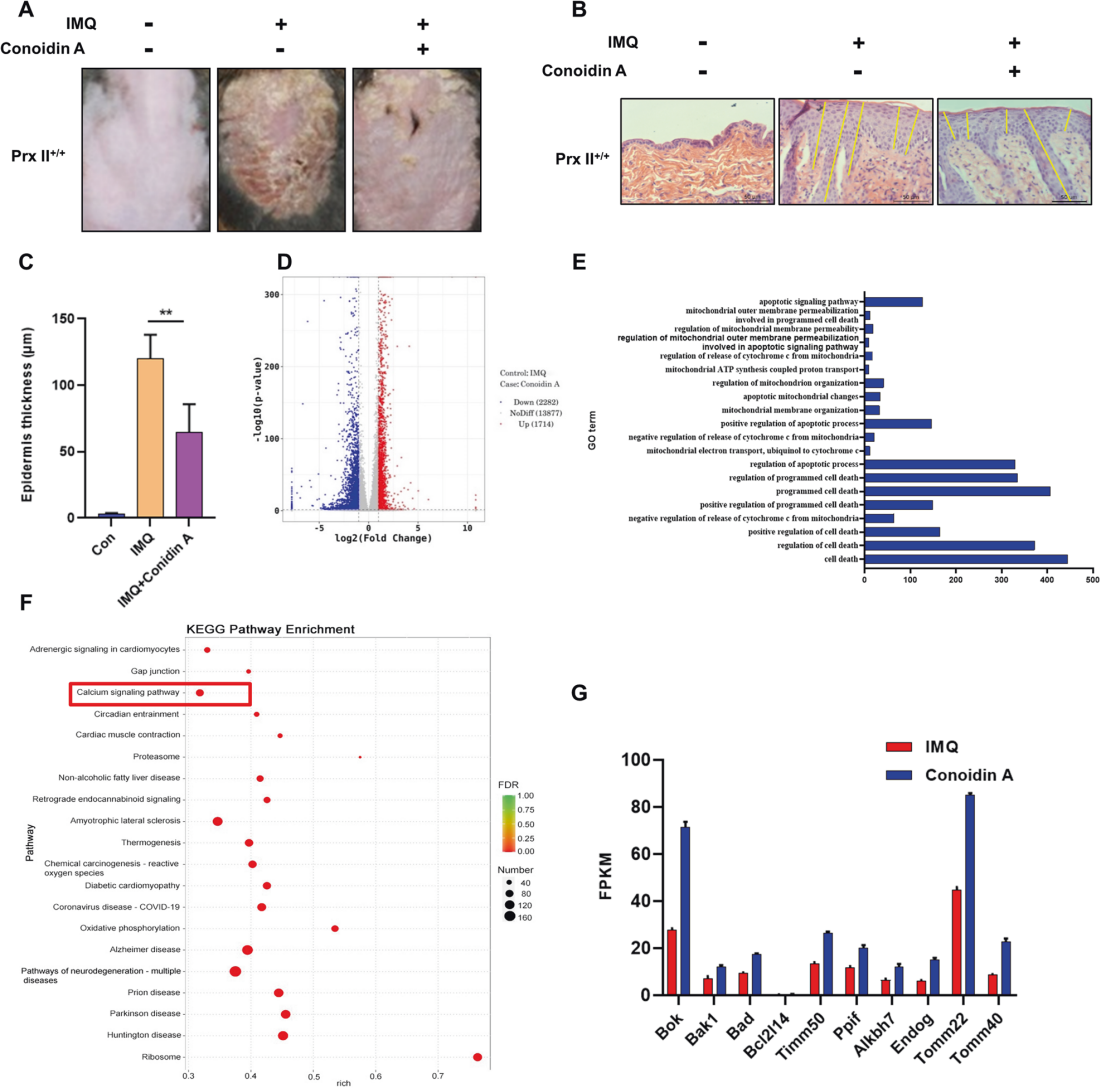
Therapeutic effect of Conoidin A on mice with psoriasis symptoms. **A** Conoidin A and IMQ were used in combination to treat the skin on the back of Prx II^+/+^ mice (*n* = 6). **B** Histological changes in the skin on the back of a mouse as assessed by H&E staining; the yellow line indicates the diameter of the skin. **C** Changes in skin thickness as compared to psoriatic mice post Conoidin A treatment. The data are represented by mean ± SEM (*n* = 6). **D** Volcano map manifestation of 3996 DEGs associated with the skin of psoriatic mice post Conoidin A treatment as determined by RNA sequencing (control vs. Conoidin A group). **E** GO terms enriched for expression in skin post Conoidin A treatment as compared to mice with psoriasis; the horizontal axis indicates number of DEGs and the vertical axis indicates enriched GO terms. **F** Kyoto Encyclopedia of Genes and Genomes (KEGG) pathways analyses. **G** The 10 genes that were significantly altered and closely associated with mitochondrial damage and mitochondria-dependent apoptosis. The data are represented by the mean ± SEM of three separate samples. (**p < 0.01).

Since the depletion of Prx II significantly reduced psoriatic symptoms in mice, Conoidin A, an inhibitor of Prx II was employed to evaluate the possibility of exploiting Prx II as a potential therapeutic target for the disease. Skin on the back of Prx II^+/+^ mice was treated with IMQ alone for 4 days, followed by combined treatment with 5 mg/kg of Conoidin A and IMQ for an additional 3 days. Mice in the Conoidin A treatment group displayed a significantly lesser degree of skin surface erythema, scaling, and psoriatic lesions as compared to those in the IMQ alone treatment group (Fig. 7A). Histological examination and statistical analysis revealed significantly thinner skin in mice that additionally received Conoidin A as compared to those that only received IMQ (Fig. 7B, C). This indicates that Prx II may serve as a potential therapeutic target for the development of effective treatments for psoriasis.

The molecular mechanisms underlying the therapeutic effects of Conoidin A were analyzed by RNA sequencing of psoriatic skin samples of mice before and after treatment with Conoidin A. The results revealed the presence of 3996 genes that were differentially expressed (Fig. 7D). GO enrichment analysis revealed significant enrichment of differentially expressed genes (DEGs) involved in cell apoptosis and mitochondrial dysfunction process (Fig. 7E). KEGG pathway analysis showed that the DEGs were mainly associated with Calcium signaling pathway (Fig. 7F). Moreover, genes associated with mitochondria-dependent apoptosis and mitochondrial damage were significantly upregulated. This suggests that Conoidin A may exert its therapeutic effects by inhibiting Prx II to regulate keratinocytes apoptosis, and may consequently serve as a key novel gene targets for the treatment of psoriatic skin lesion (Fig. 7G).

## Discussion

Psoriasis is a chronic inflammatory skin disease characterized by the appearance of red plaques comprising an obvious boundary and white scales. Histological examination of these plaques has revealed abnormal proliferation of keratinocytes, as well as immunocyte and vascular proliferation and infiltration [18]. The present study demonstrates that Prx II knock- down HaCaT cells are sensitive to H_2_O_2_ stimulation. This effect exerted was found to be mediated by the subsequent activation of the PI3K/Akt/GSK3β signaling pathway, followed by the activation of IP3R in the endoplasmic reticulum that resulted in the elevation of intracytoplasmic Ca^2+^, which finally culminated in mitochondria-dependent apoptosis. Our results conclusively demonstrate that Prx II is a key player in ROS induced mitochondria-dependent apoptosis of keratinocytes, and may therefore serve as a therapeutic target for psoriasis at the genetic level.

A comparison of the proteome of psoriatic skin with that of normal skin, revealed a significant elevation in the expression of Prx II. The protein is known to be involved in the maintenance of the redox balance, and may thus play an important role in the pathogenesis of psoriasis by regulating ROS [13]. We have demonstrated the cytoplasmic localization of Prx II, as well as its efficaciousness in the elimination ROS in dermal mesenchymal stem cells, hippocampal neurons, and L02 hepatocytes [19–22]. Further, the protein inhibits the phosphorylation of GSK3β (ser9) in hippocampal neurons and consequently inhibits cell apoptosis [19]. However, the mechanisms employed by GSK3β to exert these effects remain unclear. Our results indicated that GSK3β may regulate mitochondria-dependent apoptosis by modulating intracellular Ca^2+^ levels. ROS have been shown to play a key role in the regulation of Ca^2+^ release from the endoplasmic reticulum [23–25]. We utilized CaCl_2_ and H_2_O_2_ to treat control, and Prx II knocked down HaCaT cells, to explore the role of Prx II in exogenous and endogenous Ca^2+^ induced apoptosis of HaCaT cells. Figure 3 shows that Ca^2+^ release from the endoplasmic reticulum, which is modulated in some degree by the activity of Prx II, induces HaCaT cell apoptosis. IP3Rs are known to contain the GSK3β consensus sequence for phosphorylation at Ser1756, which activates these channels and subsequently promotes Ca^2+^ outflow from ER [26]. Pretreatment of HaCaT cells with an IP3R inhibitor indicates that GSK3β may regulate the activation of IP3R, and thereby modulate the release of Ca^2+^ from the endoplasmic reticulum into the cytoplasm, thus indirectly regulating mitochondrial damage, and apoptosis.

We have previously demonstrated that Prx II inhibits GSK3β (Ser9) activity, regulates mitochondrial damage, and thereby results in the reversal of apoptosis in mouse hippocampal neuronal cells [19]. However, the underlying mechanisms behind the regulation of GSK3β (Ser9) phosphorylation by Prx II remain unclear. Our results indicated that Prx II regulates GSK3β (Ser9) phosphorylation through the PI3K/AKT pathway. Prx II is known to contain two Cys residues, and is mainly localized to the cytoplasm. An increase in the level of intracellular ROS, is accompanied by the gradual oxidation of the Cys residues into the sulfenic form (Cys-SOH), sulfinic form (Cys-SO_2_H) and Prx-sulfonic acid, which concomitantly results in the lowering of intracellular ROS levels [27, 28]. Many studies have shown that ROS mediated PI3K/Akt Pathway activation [29–32]. However, the exact mechanism has not yet been clarified and needs further study.

The regulatory role of Prx II in psoriasis was further examined by treating a skin mouse model of psoriasis with a crystalline, cell permeable solid compound referred to as Conoidin A. The compound is known to inhibit Prx II activity by covalently binding to its cysteine residues [33]. The epidermis of the psoriasis model mice was found to be significantly thinner if they were treated with Conoidin A. This indicates that Conoidin A may improve symptoms of psoriasis by possibly targeting Prx II, which is a key regulatory factor in the onset and development of psoriatic skin lesion. RNA sequencing of skin tissue treated with either IMQ, or IMQ in combination with Conoidin A revealed that the GO terms as well as specific genes involved in cell death, and apoptosis process were significantly modified in skin tissue post Conoidin A treatment. This implies that Conoidin A may alleviate the symptoms of psoriasis via regulating the apoptosis of epidermal cells.

## Conclusion

**Fig. 8:**
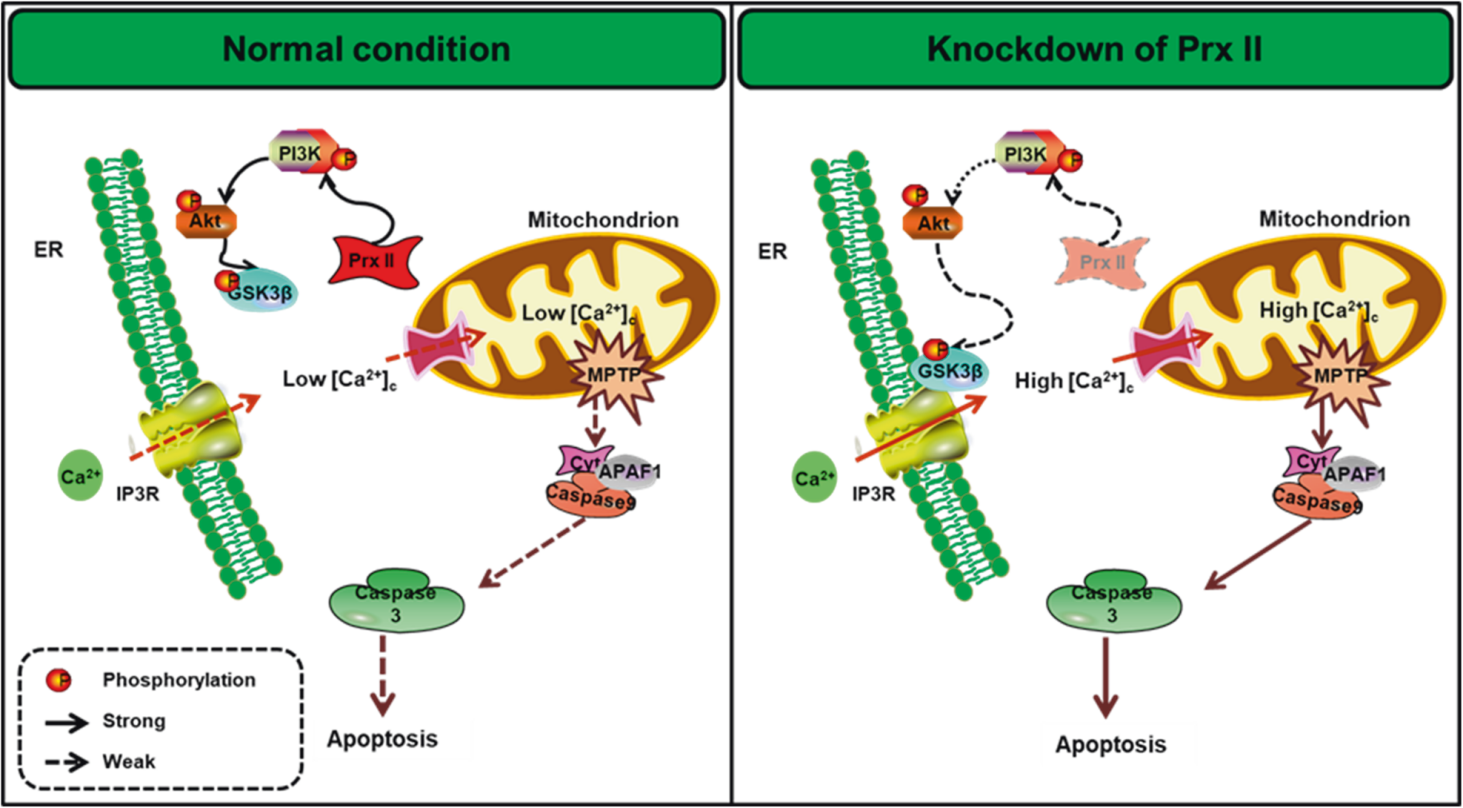
Prx II knock-down HaCaT cells are sensitive to H_2_O_2_ stimulation. This effect exerted was found to be mediated by the subsequent activation of the PI3K/Akt/GSK3β signaling pathway, followed by the activation of IP3R in the endoplasmic reticulum that resulted in the elevation of intracytoplasmic Ca^2+^, which finally culminated in mitochondria-dependent apoptosis.

In conclusion, our findings demonstrate that ROS regulate GSK3β dephosphorylation via the PI3K/Akt pathway, which in turn activates IP3R on the ER membrane to induce the release Ca^2+^ into the cytoplasm. These further results in mitochondria-dependent apoptosis in HaCaT cells. Furthermore, the inhibition of Prx II was found to improve psoriatic symptoms in a mouse model of psoriasis. These findings suggest that Prx II regulated apoptosis of keratinocytes via the PI3K/AKT/GSK3β signaling pathway (Fig. 8), Prx II may therefore serve as a gene target for the development of therapeutic options that mediate apoptosis in order to treat psoriasis.

## Materials and methods

### Cell culture

Human immortalized keratinocytes (HaCaT) were procured from Bo Gu (Shanghai, China), and cultured in 1640 medium supplemented with 10% FBS and 100 μg/mL penicillin and streptomycin at 37 °C in air supplemented with 5% CO_2_. Cells were sub-cultured after treatment with 0.25% Trypsin–EDTA once they reached coverage of 90%.

### Experimental animal

The mice were exposed to 12 h dark/light cycles, with free access to food and water at 20–22 °C ambient temperature and 50–60% humidity. All experiment treatments conformed to the guidelines laid down by the Animal Care and Use Committee. Thirty male 129/SVJ mice 4–8 weeks were used in the experiments. An area of 2 × 2 cm^2^ on the back of these mice was cleared of hair. The mice were then randomly divided into five groups, namely, the Prdx2^+/+^ (wild) control mice, Prdx2^–/–^ (knockout) control mice, Prdx2^+/+^ mice treated with Imiquimod (IMQ), Prdx2^–/–^ mice treated with IMQ, and Prdx2^+/+^ as well as Prdx2^–/–^ mice treated with Conoidin A and IMQ. Vaseline and IMQ were topically applied for 7 days to the backs of control mice and those that received only IMQ, respectively. Mice that received combination therapy with Conoidin A and IMQ were topically administered IMQ for the first three days, followed by Conoidin A and IMQ for the next four days. After a total of 8 days post application of the drugs, blood samples were collected from the orbit, and the levels of cytokines. Mice were subsequently sacrificed, and skin samples from their backs were processed for histological evaluation.

### Cell viability assay

A total of 8 × 10^3^ HaCaT cells were seeded in 96-well plates. Cells were subsequently treated with varying concentrations of H_2_O_2_ (0–20 μM), CaCl_2_ (0–2 mM), TNF-α (0–100 ng/mL), and IL-22 (0–100 ng/mL). Cell viability was assessed by the MTT assay. Absorbance was measured at 490 nm after completion of the assay.

### Flow cytometry analysis

A total of 4 × 10^5^ HaCaT cells were plated per well in 6-well plates and cultured for 24 h and treated with varying concentrations of H_2_O_2_ 20 μM. The cells were incubated respectively with dihydroethdium (DHE) dye, Fluo3-AM, and Annexin V-PE in cell binding buffer. The level of intracellular ROS, Ca^2+^ and the level of apoptosis were detected respectively by flow cytometry. Each experiment was performed in triplicates.

### Western blot analysis

A total of 4 × 10^5^ HaCaT cells were plated per well in 6-well plates and cultured for 24 h and treated with varying concentrations of H_2_O_2_ 20 μM. Total protein HaCaT cell lysates were separated by SDS AGE using a 12% SDS polyacrylamide gel. The separated proteins were subsequently transferred to a nitrocellulose membrane, which was blocked with 5% skim milk prepared in TBST. The blocked membrane was incubated overnight at 4 °C with anti-Prx II (cat. no. LF-MA0144, AbFrontier), anti-Bax (cat. no. E-AB-13814, Elabscience Biotechnology), anti-Bcl2 (cat. no. E-AB-60788, Elabscience Biotechnology), anti-cleaved-Caspase 3/Caspase3 (cat. no. ab184787, abcam), anti-β-actin (cat. no. sc-47778, Santa Cruz Biotechnology), anti-pGSK3β(Ser9) (cat. no. AF5830, Beyotime Institute of Biotechnology), anti-GSK3β (cat. no. AF1543, Beyotime Institute of Biotechnology), and anti-pAKT (cat. no. sc-7985-R, Santa Cruz Biotechnology), anti-AKT(cat. no. sc‑8044, Santa Cruz Biotechnology), anti-pPI3K (cat. no. AF3242, Affinity Biosciences), and anti-PI3K (cat. no. 4257s, Cell Signaling Technology). The membrane was subsequently washed and incubated with mouse and rabbit IgG secondary antibodies (cat. no. D110087 and D110058; Shanghai Sangon Biotech Co.) at room temperature for 2 h. Post washing with TBST to remove excess antibodies, a chemiluminescence detection system was used to identify the presence of the probed proteins. Image J software was used to quantify the band intensity.

### Statistical analysis

All data are presented as mean ± SEM values from at least three independent experiments. Repeated measures one-way ANOVA and Tukey’s honest significant difference tests were used to compare groups. A *p* value <0.05 was considered statistically significant. SPSS Statistics v. 25 software (IBM, Armonk, NY, USA) was used for statistical analysis.

## Data Availability

The original contributions presented in the study are included in the article/Supplementary Material. Further inquiries can be directed to the corresponding authors.
